# Chronic Inflammatory Demyelinating Polyneuropathy: A Narrative Review of a Systematic Diagnostic Approach to Avoid Misdiagnosis

**DOI:** 10.7759/cureus.76749

**Published:** 2025-01-01

**Authors:** Rodolfo M Roman-Guzman, Adriana P Martinez-Mayorga, Louis D Guzman-Martinez, Ildefonso Rodriguez-Leyva

**Affiliations:** 1 Neurology, Facultad de Medicina, Universidad Autonoma de San Luis Potosi, San Luis Potosi, MEX; 2 Neurology, Hospital Central Dr. Ignacio Morones Prieto, San Luis Potosi, MEX

**Keywords:** atypical-cidp, chronic inflammatory demyelinating polyneuropathy (cidp), clinical phenotypes, electrophysiological criteria, inflammatory neuropathies, typical-cidp

## Abstract

Chronic inflammatory demyelination polyradiculoneuropathy (CIDP) is a rare autoimmune neuropathy generated by cellular and humoral immune responses. Its course can be chronic, progressive, monophasic, or relapsing-remitting. Misdiagnosis and inappropriate therapy are common in CIDP. Given the scarcity of integrative information, we aimed to briefly summarize the epidemiology, pathophysiology, clinical phenotypes, diagnostic tools, and diagnostic criteria and provide a systematic diagnostic approach.

We reviewed articles on Medline (PubMed) from 2018 to 2023, using Google Scholar to summarize the topics. The results are presented as a narrative review, in accordance with recommendations of the Scale for the Assessment of Narrative Review Articles (SANRA) guidelines.

The included evidence showed that CIDP is a challenging neuropathy to diagnose and treat. Pathologic factors initiating typical CIDP and atypical CIDP are still clearly unknown. CIDP is diagnosed using the European Federation of Neurological Societies/Peripheral Nerve Society (EFNS/PNS) criteria, which combine clinical features with electrophysiological evidence of demyelination. However, some patients need to fulfill the requirements. Another challenge is monitoring the disease progression and recognizing patients who do not respond to evidence-based first-line therapy to individualize their treatment.

Based on the evidence, we conclude that 2021 EFNS/PNS guidelines allow for a more accurate diagnosis and treatment of CIDP and its variants. New diagnostic tools and molecular approaches are helpful in the diagnosis process but cannot replace clinical and electrodiagnostic criteria.

## Introduction and background

Chronic inflammatory demyelination polyradiculoneuropathy (CIDP) is the most common form of chronic inflammatory neuropathy. It is a rare autoimmune neuropathy generated by cellular and humoral immune responses. It can present as a chronic progressive, monophasic, or relapsing-remitting form, with proximal and distal muscle weakness that develops for at least two months [1,2]. 

The diagnosis of CIDP is mainly based on clinical and electrophysiological criteria used by the European Federation of Neurological Societies/Peripheral Nerve Society (EFNS/PNS) [3]. Thus, CIDP can be classified into typical CIDP and atypical variants, such as distal acquired-demyelinating polyneuropathy (DADS), multifocal-acquired demyelinating sensory and motor polyneuropathy (MADSAM), also referred to as Lewis-Sumner syndrome (LSS), and acute-onset CIDP (A-CIDP). However, some patients, such as those with severe early axonal damage, do not fulfill the criteria [4].

Misdiagnosis in CIDP and inappropriate therapy is still widespread, particularly in those classified as CIDP variants [3,5-7]. In a cross-sectional quantitative survey study performed in the United States, 87% of the respondents declared they needed to become more familiar with the 2021 EFNS/PNS guidelines. Further, variability in treatment approaches existed regarding the dose of intravenous immunoglobulin (IVIg) used, the length of IVIg therapy before determining response, the outcome measures used to determine IVIg response, and the protocol for weaning off therapy [8]. Given the scarcity of integrative information, this article briefly summarizes the epidemiology, pathophysiology, clinical phenotypes, diagnostic tools, and diagnostic criteria to provide a systematic diagnostic approach to avoid misdiagnosis.

## Review

A general search on PubMed and Google Scholar was done using the mesh terms (CIDP) AND (Typical CIDP) AND (Atypical CIDP) AND (Diagnosis), including articles from 2018 to 2023. We selected articles written in English and considered only systematic reviews, guidelines, cohort studies, and clinical trials related to adult patients. 

The results are presented as a narrative review, in accordance with the recommendations of the Scale for the Assessment of Narrative Review Articles (SANRA) guidelines. The thematic analysis of the literature search helped identify several key topics for structuring the review. These topics include the epidemiology of CIDP, which examines the prevalence and demographic distribution of the condition. Another critical area of focus is the clinical phenotypes, which describe how CIDP can manifest among patients. The pathophysiology of CIDP is also discussed, highlighting the immune-mediated mechanisms involved in the disease's progression.

Diagnostic evaluation is significant in detailing the approaches used to confirm the condition. The supportive diagnosis is further explored, emphasizing the role of additional tests in reinforcing the diagnosis. Lastly, the review covers the treatment of CIDP, describing various therapeutic strategies and their effectiveness.

Epidemiology

According to a meta-analysis in 2019, the prevalence of CIDP ranges from 0.67 to 7.7 per 100,000, with different prevalence rates in various geographical regions [9]. CIDP is most common in men and usually occurs between 40 and 60 years of age; however, it can also affect children [2,3]. 

More than half of the patients are considered to have a typical variant. Nevertheless, the frequency of presentation of atypical variants differs in the literature. The Italian CIDP database study group analyzed 460 patients with CIDP, and they found that 82% of them had typical CIDP and the remaining 18% had atypical CIDP; the atypical presentations of CIDP included DADS (7%), LSS (4%), pure motor CIDP (4%), and pure sensory CIDP (3.5%). In a Japanese study, 100 patients with CIDP were classified as having typical CIDP (60%); the rest had MADSAM (34%), DADS (5%), or pure sensory CIDP (1%). 

Crude CIDP incidence rates vary between 0.15 to 10.6 cases per 100,000 person-years [9]. 

Clinical phenotypes 

CIDP diagnosis implies a combination of clinical, electrodiagnostic, and laboratory features that resemble alternative diagnoses and even syndromes that fulfill the criteria for typical CIDP [10]. In general, CIDP results in damage to peripheral nerve myelin, and heavily myelinated fibers are the most susceptible to injury. Patients typically present with numbness, weakness, and sensory ataxia. However, the clinical presentation varies from one patient to another [11]. 

*Typical CIDP* 

More than half of CIDP patients present with a progressive course of paraesthesia, weakness in the distal limbs, and difficulty walking for more than eight weeks, but can be relapsing-remitting. It is more common in males and can occur at any age, but it is most common between 40 and 60 years. The clinical examination shows progressive symmetric proximal and distal muscle weakness, sensory loss, and decreased or absent deep tendon reflexes [3,4,11].

Treatment response: IVIg, corticosteroids, and plasma exchange are effective [11]. 

A-CIDP

Typical CIDP may present acutely (A-CIDP) in up to 13% of patients, who rapidly progress within four weeks and initially may be diagnosed with Guillain-Barré syndrome (GBS). A-CIDP patients differentiate from those with GBS because they continue to deteriorate more than eight weeks after the onset or even relapse at least three times after initial improvement. Furthermore, A-CIDP patients can walk independently, are less likely to have facial weakness or respiratory or autonomic nervous system involvement, and are more likely to have sensory signs. It is essential to know that there are no specific clinical features or laboratory tests that can distinguish GBS from A-CIDP in the acute stage of the disease [3,11].

Treatment response: The practical treatment possibilities are IVIg, corticosteroids, and plasma exchange [11]. 

*Sensory CIDP* 

Sensory symptoms are predominant, including ataxia, but without weakness and in a polyneuropathic distribution, excluding a length-dependent pattern (included under DADS). Patients can also present neuropathic pain, fatigue, tremor, and facial sensory symptoms. The clinical course can be either relapsing or progressive and, more rarely, monophasic [12]. The 2021 EFNS/PNS criteria subclassify pure sensory CIDP in a subform with normal motor nerve conduction studies (“pure sensory CIDP”) and another with abnormal motor nerve conduction studies (“sensory-predominant CIDP”) [3,13].

Treatment response: IVIg, corticosteroids, and plasma exchange are effective [11].

Chronic Immune Sensory Polyradiculopathy (CISP)

It is not considered part of the CIDP spectrum as per the 2021 EFNS/PNS criteria. CISP is generally regarded as pure sensory CIDP, with its distinct features being the selective involvement of the preganglionic root, as evidenced by normal sensory nerve conduction studies, increased cerebrospinal fluid (CSF) protein levels, and thickened spinal roots at MRI [13]. 

Treatment response: IVIg and corticosteroids are effective [11].

CISP-Plus

It is a recently described variant in which the disease extends beyond dorsal roots to involve motor and postganglionic sensory nerve fibers, resulting in mild distal weakness and mild abnormalities in nerve conduction studies. Its symptoms and response to therapy seem very similar to those of CISP [13]. 

Although experts traditionally proposed all these rare forms as part of the CIDP spectrum, the 2021 EFNS/PNS criteria only mention CISP. They specified that it cannot still be considered CIDP as there is insufficient evidence to determine if it is demyelinating or related to sensory CIDP [3,9].

Motor CIDP 

Motor symptoms are predominant, including weakness, cramps, fatigue, and tremors, in a polyneuropathic distribution. Motor cranial nerve palsy could be present [12]. The 2021 EFNS/PNS guidelines subclassifies pure motor CIDP in a subform with normal sensory nerve conduction studies (“pure motor CIDP”) and in another with abnormal sensory nerve conduction studies (“motor-predominant CIDP”) [3,13]. 

Treatment response: IVIg is effective; patients' conditions may deteriorate after corticosteroids [11]. 

MADSAM

The first description of an asymmetric CIDP variant was LSS, a chronic asymmetric sensorimotor neuropathy with focal involvement of individual nerves with persistent conduction block [14]. Since this first report, multiple other terms have been introduced. However, in the actual guidelines, MADSAM is preferred.

It usually affects the upper limbs first. Lower limbs may become involved later or sometimes are affected from the onset. Cranial nerves, including oculomotor, trigeminal, facial, vagal, and hypoglossal nerves, are probably more frequently involved than in other CIDP forms [3]. Additionally, it can be presented with sensory symptoms, with or without weakness, in a multifocal distribution. Symptoms may start anywhere in the body [12]. 

Treatment response: IVIg, corticosteroids, and plasma exchange are adequate, with an overall response rate of 70%. Rituximab could be considered as a second-line agent in refractory cases [11].

DADS

DADS is defined by a symmetrical presentation of sensory and sensorimotor symptoms starting distally in the lower limbs. There is no proximal limb nor cranial nerve involvement [15]. Patients present with sensory loss in the distal upper and lower limbs and gait instability. Weakness may occur and is usually distally accentuated in the lower more than upper limbs [3]. Other possible symptoms include ataxia, neuropathic pain, cramps, fatigue, autonomic symptoms, and tremors. Upper-limb distal sensory or sensorimotor symptoms and signs may occur later (at least one year from onset) [12].

DADS could be divided into idiopathic DADS (DADS-I) and DADS with elevated monoclonal protein (DADS-M). Approximately 50-70% of patients with DADS-M have antibodies against myelin-associated glycoprotein (MAG). Distal neuropathy with an IgM paraprotein and anti-MAG antibodies, anti-MAG neuropathy, is considered outside the span of CIDP as most of these patients have specific electrodiagnostic and pathologic findings and do not respond to IVIg or corticosteroids. 

Treatment response: Patients with DADS-I respond similarly (reaching 70-80% response) to conventional treatment with IVIg, corticosteroids, and plasma exchange. On the other hand, rituximab is preferred for patients with DADS-M who need hematological follow-up [15]. 

An overview of the phenotypes of typical CIDP and atypical CIDP variants is provided in Table 1.

**Table 1:** Comparison of features of CIDP and its variants CIDP, Chronic inflammatory demyelination polyradiculoneuropathy; A-CIDP, acute-onset CIDP; CSF: Cerebrospinal fluid; DADS, Distal acquired demyelinating and symmetric neuropathy; dmL, Distal motor latencies; GBS, Guillain-Barré syndrome; IVIg, Intravenous immunoglobulins, LLS, Lewis-Summer syndrome; MADSAM, Multifocal acquired demyelinating sensory and motor neuropathy; MAG, Myelin-associated glycoprotein; NCV, Nerve conduction velocity; NF155, Neurofascin 155; CNTN1, Contactin 1 [3-5]

CIDP variant	Percentage of CIDP cases	Clinical features	Treatment response	Electrophysiological hallmarks	Additional information
Typical CIDP					
Typical CIDP	>50%	Symmetric proximal and distal weakness, sensory loss, areflexia	IVIg, corticosteroids, and plasma exchange effective	Demyelination	Clinical nadir reached after eight weeks
A-CIDP	Around 13%	Symmetric proximal and distal weakness, sensory loss, areflexia	IVIg, corticosteroids, and plasma exchange effective	Demyelination	Progress within four weeks. 5% of patients initially diagnosed with GBS are later reclassified as A-CIDP; might resemble patients with NF155 and CNTN1 antibodies
Atypical CIDP variants					
Sensory CIDP	5-15%	Symmetric sensory- predominant presentation, sensory ataxia, generalized hyporeflexia/ areflexia	IVIg, corticosteroids, and plasma exchange effective	Normal motor NVC	Distinguished from idiopathic sensory polyneuropathy by early ataxia, younger age, early upper extremity symptoms
Motor CIDP	4-10%	Symmetric proximal and distal motor deficits	IVIg effective; patients’ condition might deteriorate after corticosteroids	May have electrophysiologic evidence of sensory involvement	Motor CIDP with sensory conduction abnormalities in two nerves is diagnosed as motor-predominant CIDP.
MADSAM	8-15%	Asymmetric distal more than proximal sensory and motor deficits; affects upper more than lower extremities	IVIg, corticosteroids, and plasma exchange effective	Multifocal distributed sensory and motor symptoms and nerve conduction studies frequently show conduction blocks	Also known as LSS. May evolve into typical CIDP
DADS	2-10%	Symmetric distal more than proximal, sensory more than motor deficits; affects lower more than upper extremities	With MAG antibodies, treatment is ineffective, although rituximab response was reported in some series; without MAG antibodies, response to IVIg, corticosteroids, and plasma exchange.	Often associated with abnormally increased dmL	May be associated with an IgM monoclonal gammopathy; may have MAG antibodies. Considered DADS-CIDP in the absence of monoclonal gammopathy and MAG antibodies

Pathophysiology

CIDP is considered an immune-mediated disorder; contrary to GBS, antecedent infections are rarely reported in CIDP [11]. 

Experimental evidence on passive and active animal transfer models, active immunization with nerve components, and response to immunosuppressive treatment, IVIg, and plasmapheresis suggest that dysfunctional acquired immune responses may play a pivotal role in the pathogenesis of CIDP [16]. There is evidence of involvement of auto-reactive T cells, B cells, soluble factors in nerve tissue including inflammatory cytokines and chemokines, antibodies against various nerve glycolipid and glycoprotein structures, and increased concentrations of complement factors (e.g., C5a, soluble terminal complement complex) [17].

There is also evidence of increased expression levels of the activating Fcγ receptor I (FcγRI) on monocytes and a reduced expression level of the inhibitory Fcγ receptor IIB (FcγRIIB) on naive and memory B cells, as well as on monocytes, in the blood of treatment-naive patients with CIDP. This disturbed Fcγ receptor regulatory system in subjects with CIDP was partly restored after IVIg therapy. Additional evidence of an underlying immune-mediated mechanism is the improvement after IVIg, corticosteroids, and plasma exchange in patients with CIDP [4,18,19].

It is important not to confuse the immunologic process of autoimmune neuropathies (e.g., antibodies against contactin-associated protein 1 (Caspr1), contactin 1 (CNTN1), neurofascin (NF) 186, and NF155), anti-MAG neuropathy, polyneuropathy-organomegaly-endocrinopathy-M-protein-skin changes (POEMS) syndrome, and multifocal motor neuropathy (MMN) because they are not included as part of the CIDP syndrome [20,21].

**Figure 1:**
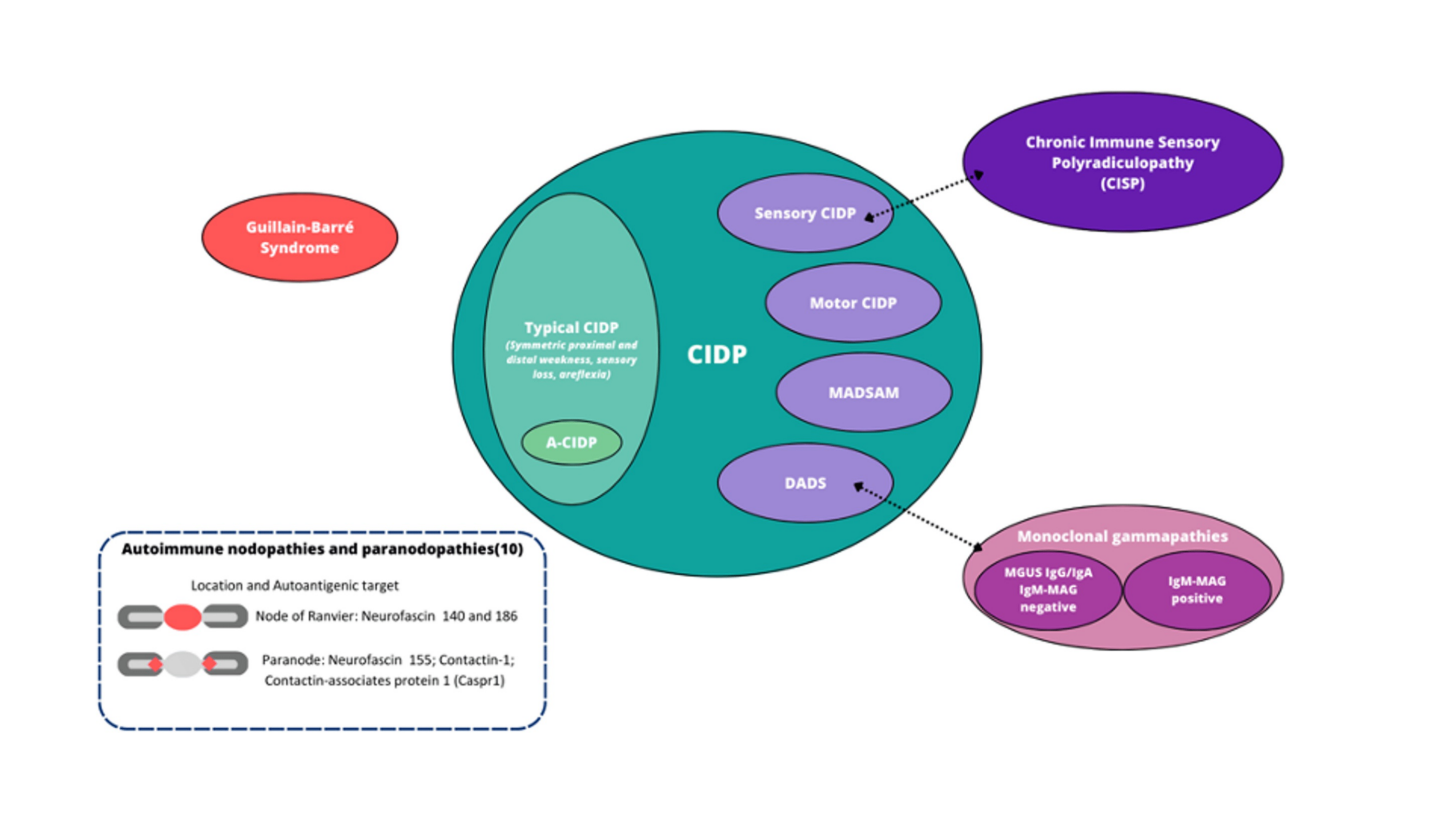
Spectrum of CIDP based on the 2021 second revision of the European Academy of Neurology/Peripheral Nerve Society guidelines CIDP, Chronic inflammatory demyelination polyradiculoneuropathy; DADS, Distal acquired demyelinating and symmetric neuropathy; MADSAM, Multifocal acquired demyelinating sensory and motor neuropathy; A-CIDP, acute-onset CIDP Figure credits: Rodolfo M. Roman-Guzman

Figure 1 clarifies changes in the spectrum of CIDP based on the 2021 second revision of the European Academy of Neurology/Peripheral Nerve Society (EAN/PNS) guidelines. 

Diagnostic evaluation

All forms of CIDP have clinical points in common; however, as explained above, the pathogenic mechanism and clinical presentation may differ between variants, and these unique characteristics in each variant modify the differential diagnosis. 

The 2021 EFNS/PNS guidelines include differential diagnostic considerations for each variant and have reduced the levels of diagnostic certainty to two: “CIDP” and “possible CIDP.” As noted, recent guidelines avoid using the term “definite CIDP” due to the lack of a gold standard for diagnosis and because of empirical evidence showing that the sensitivity and specificity of electrodiagnostic criteria to define “probable CIDP” and “definite CIDP” (as used previously) do not significantly differ [3,13]. 

The application of the 2021 EAN/PNS criteria has been evaluated in a retrospective study on 120 consecutive patients with a diagnosis of “suspected CIDP." The EAN/PNS criteria sensitivity for “CIDP” was 83.3%. The sensitivity for “CID” or “possible CIDP” was 93.3%. Specificity was 94% for “CIDP” and 79% for “possible CIDP” [22].

Several investigations are strongly recommended for diagnosing typical CIDP and its variants. These include electrodiagnosis involving motor and sensory nerve conduction studies and serum and urine monoclonal protein detection through immunofixation. Additionally, fasting blood glucose levels should be assessed. A complete laboratory blood count and renal and liver function evaluations are also necessary. As this text describes, other investigations would be indicated if the differential diagnosis process requires them. 

Table 2 presents the electrodiagnostic motor nerve conduction criteria, while Table 3 describes the sensory nerve conduction criteria. It is also important to mention that patients not reaching the cut-off electrophysiological demyelinating values, such as patients with early disease, can still fulfill the requirements in later examinations [23]. Therefore, if clinical suspicion is high, a second check is advised. 

**Table 2:** Motor nerve conduction criteria Criteria are based on electrophysiological parameters for diagnosing demyelination disorders. Consider combining weak criteria with clinical findings for robust diagnosis. Ensure technical artifacts, such as temperature effects, are excluded during nerve conduction studies. ULN, Upper limit of normal; LLN, Lower limit of normal; CMAP, Compound muscle action potential; LFF, Low-frequency filter

Criteria	Details
Strong support for demyelination	At least one of the following:
	Prolongation of distal motor latency ≥50% above ULN in two nerves (excluding median neuropathy at the wrist due to carpal tunnel syndrome)
	Reduction of motor conduction velocity ≥30% below LLN in two nerves
	Prolongation of F-wave latency ≥20% above ULN in two nerves (≥50% if distal negative peak amplitude CMAP <80% of LLN)
	Absence of F waves in two nerves (if these nerves have distal negative peak CMAP amplitudes ≥20% of LLN) + ≥1 other demyelinating parameter in ≥1 other nerve
	Motor conduction block: ≥30% reduction in proximal maximum negative CMAP amplitude relative to distal, excluding tibial nerve, and distal maximum negative CMAP amplitude ≥20% of LLN in two nerves or one nerve + ≥1 another parameter
	Abnormal temporal dispersion: >30% increase in duration between proximal and distal negative peak CMAP (at least 100% in the tibial nerve) in ≥2 nerves
	Distal duration of CMAP prolongation in ≥1 nerve + ≥1 other demyelinating parameter in ≥1 other nerve
LFF values	(2 Hz): Median >8.4 ms, Ulnar >9.6 ms, Peroneal >8.8 ms, Tibial >9.2 ms
	(5 Hz): Median >8.0 ms, Ulnar >8.6 ms, Peroneal >8.5 ms, Tibial >8.3 ms
	(10 Hz): Median >7.8 ms, Ulnar >8.5 ms, Peroneal >8.3 ms, Tibial >8.2 ms
	(20 Hz): Median >7.4 ms, Ulnar >7.8 ms, Peroneal >8.1 ms, Tibial >8.0 ms
Weak support for demyelination	As in a single nerve

**Table 3:** Sensory nerve conduction criteria Skin temperature should be maintained at a minimum of 33°C at the palm and 30°C at the external malleolus. Since these criteria do not allow the identification of mean reference values compatible with sensory nerve demyelination, sensory CIDP can only be a possible diagnosis based on clinical and electrophysiological criteria. The amplitude of the sural nerve action potential decreases with age, so age-dependent reference values are recommended from 60 years of age onwards. CIDP, Chronic inflammatory demyelinating polyradiculoneuropathy; LLN, Lower limit of normal; SNAP, Sensory nerve action potential

Sensory nerve conduction criteria
(1) CIDP - Sensory conduction abnormalities (prolonged distal latency, reduced SNAP amplitude, or slow conduction velocity outside normal limits) in two nerves.
(2) Possible CIDP - As in (1) but in a single nerve. Sensory CIDP with normal motor nerve conduction studies needs to meet: (a) Sensory nerve conduction velocity <80% of LLN (for SNAP amplitude >80% of LLN) or <70% of LLN (for SNAP amplitude <80% of LLN) in at least two nerves (median, ulnar, radial, sural nerve); or (b) Sural sparing pattern (abnormal median or radial SNAP with normal sural nerve SNAP) (excluding carpal tunnel syndrome).

Typical CIDP 

Clinical criteria: The clinical criteria for a typical CIDP require the following characteristics: progressive or relapsing, symmetric, proximal, and distal muscle weakness in both the upper and lower limbs, sensory involvement in at least two limbs, and absence or reduced myotatic reflexes in all limbs. Symptoms should develop over a minimum of eight weeks.

Electrodiagnostic criteria: To confirm the diagnosis of typical CIDP, we must try to find abnormalities in at least two motor nerves that fulfill the motor conduction criteria. If the criteria are met in only one nerve, the diagnosis may be considered possible typical CIDP. Sensory conduction abnormalities are required in at least two nerves. In cases where patients fulfill the clinical criteria but not the minimal electrodiagnostic criteria, the diagnosis of possible typical CIDP may still be made if there is objective improvement following treatment with IVIg, corticosteroids, or plasma exchange, provided that at least one additional supportive criterion is also fulfilled.

Differential diagnosis: Multiple conditions to consider in the differential diagnosis include amyloid light chain (AL) amyloidosis, hereditary amyloid transthyretin (ATTRv) polyneuropathy, chronic ataxic neuropathy with ophthalmoplegia, M-protein agglutination, and disialosyl antibodies (CANOMAD). Other potential conditions include Guillain-Barré-Strohl syndrome, liver neuropathy, AIDS-HIV-related neuropathy, multiple myeloma, osteosclerotic myeloma, POEMS syndrome, uremic neuropathy, and vitamin B12 deficiency, whether actual or functional (such as due to nitrous oxide poisoning).

*Sensory CIDP* 

Clinical criteria: These include sensory symptoms and signs without motor involvement. Long-term follow-up studies have shown that sensory CIDP is frequently a transient clinical stage that precedes the presentation of weakness in about 70% of patients.

Electrodiagnostic criteria: Sensory CIDP must fulfill sensory conduction criteria, and motor conduction must be normal in at least four nerves (median, ulnar, peroneal, and tibial) to confirm the clinical diagnosis. The maximum diagnostic certainty is possible sensory CIDP.

Sensory CIDP with motor conduction criteria fulfilled in one nerve is diagnosed as possible sensory-predominant CIDP. If motor conduction criteria are fulfilled in two nerves, the diagnostic certainty increases to sensory-predominant CIDP. 

Differential diagnosis: The differential diagnosis for sensory CIDP includes several conditions: cerebellar ataxia, neuropathy, vestibular areflexia syndrome (CANVAS), CISP, and dorsal column lesions caused by syphilis, paraneoplastic conditions, copper deficiency, or vitamin B12 deficiency. Other considerations include hereditary sensory neuropathies, idiopathic sensory neuropathy, sensory neuronopathy, and toxic neuropathies, which may be due to factors like chemotherapy or vitamin B6 toxicity.

Motor CIDP

Clinical criteria: These include motor symptoms and signs without sensory involvement.

Electrodiagnostic criteria: To confirm the clinical diagnosis of motor CIDP, motor conduction abnormalities must be present in at least two nerves, and sensory conduction should remain normal in at least four nerves, specifically the median, ulnar, radial, and sural nerves. If motor conduction abnormalities are found in only one motor nerve, a diagnosis of possible motor CIDP can be considered.

Differential diagnosis: The differential diagnosis for motor CIDP includes hereditary motor neuropathies, such as distal hereditary motor neuropathies, spinal muscular atrophy, and porphyria. Other conditions to consider are inflammatory myopathies, motor neuron disease, and neuromuscular junction disorders, such as myasthenia gravis and Lambert-Eaton syndrome.

DADS

Clinical criteria: These include distal sensory loss with muscle weakness, predominantly in the lower limbs. 

Electrodiagnostic criteria: Motor conduction criteria fulfillment is necessary for at least two upper limb nerves to confirm the clinical diagnosis of distal CIDP. The distal negative peak compound muscle action potential (CMAP) amplitude should be at least 1 mV. When criteria are fulfilled in two lower limb nerves but not upper limb nerves or if criteria are fulfilled in only one upper limb nerve, the maximum diagnostic certainty is possible distal CIDP. Additionally, sensory conduction abnormalities must be present in at least two nerves.

Differential diagnosis: Conditions that must be considered in the differential diagnosis include Anti-MAG IgM neuropathy, diabetic neuropathy, hereditary neuropathies (e.g., Charcot-Marie-Tooth disease (CMT) 1, CMTX1, CMT4, metachromatic leukodystrophy, Refsum disease, adrenomyeloneuropathy, ATTRv polyneuropathy), POEMS syndrome, and vasculitic neuropathy.

Multifocal and Focal CIDP

Clinical criteria: Multifocal CIDP presents with sensory loss and muscle weakness in a multifocal pattern. This pattern is usually asymmetric and predominantly affects the upper limbs, involving more than one limb.

Electrodiagnostic criteria: Motor conduction criteria fulfillment is required in at least two nerves in more than one limb to support the clinical diagnosis of multifocal CIDP and in at least two nerves in one limb to diagnose focal CIDP. When criteria are fulfilled in only one nerve, the maximum diagnostic certainty is possible multifocal or focal CIDP.

Sensory conduction alterations must be present in at least two nerves of the affected limbs to diagnose multifocal or focal CIDP and in one nerve of the affected limb to diagnose possible focal CIDP.

Differential diagnosis: The differential diagnosis includes diabetic radiculopathy or plexopathy, entrapment neuropathies, genetic or hereditary neuropathy with liability to pressure palsies (HNPP), multifocal motor neuropathy (MMN), neuralgic amyotrophy, and peripheral nerve tumors (such as lymphoma, perineurium, schwannoma, and neurofibroma). Vasculitic neuropathy, such as mononeuritis multiplex, should also be considered.

Supportive criteria diagnosis based on additional tests

When the clinical and electrodiagnostic criteria provide the only evidence for a possible CIDP, the following additional tests can be performed to support the diagnosis:

*Response to Treatment* 

The 2021 EFNS/PNS guidelines recognize an objective response to immunomodulatory treatments, including IVIg, plasma exchange, and corticosteroids, supporting the clinical diagnosis of CIDP. This applies to patients for whom clinical, electrodiagnostic, and other supportive criteria allow only a diagnosis of possible CIDP [3,20]. An objective response to treatment is defined by improvement on at least one disability and one impairment scale.

Disability measures: Improvement can be assessed using disability scales. An increase of at least four centile points on the Inflammatory Rasch-Built Overall Disability Scale (I-RODS) indicates improvement [24]. Similarly, a decrease in at least one point on the Inflammatory Neuropathy Cause and Treatment (INCAT) disability scale also reflects improvement [20].

Impairment measures: Impairment can be evaluated through multiple measures. A change of +2 to +4 points on the Medical Research Council (MRC)-Sum Score (range: 0-60) indicates improvement [20]. The modified INCAT Sensory Sum Scale (mISS) shows improvement with at least a two-point reduction, and higher values may enhance diagnostic specificity [20].

Grip strength is another key metric. For the Martin vigorimeter, an increase of 8-14 kPa reflects improvement, and higher values may improve diagnostic specificity. For the Jamar handgrip dynamometer, an increase of at least 10% is significant, notably when values are averaged over three consecutive days, enhancing diagnostic specificity [25].

If patients demonstrate an objective response to treatment, the likelihood of a CIDP diagnosis increases. However, some patients may not respond to at least one of the three proven effective treatments and still have CIDP. Before considering alternative immunosuppressive treatment strategies, additional testing is required to rule out other disorders that mimic CIDP.

*Nerve Ultrasound* 

The 2021 EFNS/PNS guidelines suggest that ultrasound should be used only in adult patients (there is no evidence to support ultrasound in pediatric patients). Normative values for the cross-sectional area of the median nerve are available, with nerve enlargement being shown by values of >10 mm^2^ at the forearm, >13 mm^2^ at the upper arm, >9 mm^2^ for the inter scalene (trunks), and >12 mm^2^ for nerve roots [3].

In a recent study of CIDP patients (40 with typical and 40 with atypical CIDP), it was found that increased muscle echo intensity, reflecting fibrosis and a fatty infiltration due secondary to axonal damage, correlated to clinical muscular strength and dysfunction in a large cohort of CIDP patients. Alterations of echo intensity occur in typical and atypical CIDP patients and are especially pronounced in the distal leg muscles. However, further studies are needed to address whether muscular echo intensity reflecting axonal damage can be a prognostic marker in CIDP [26]. 

MRI 

The 2021 EFNS/PNS guidelines suggest not using MRI in adult patients to diagnose CIDP except in fulfilling diagnostic criteria for a possible CIDP but not CIDP and mention that there is currently no evidence to support MRI in pediatric patients [3]. It is mainly used when nerve ultrasound is not available or when nerve ultrasound results are non-contributory, and the classical sign to look for is nerve enlargement and increased signal intensity of nerve roots on T2-weighted MRI sequences (DIXON/STIR, coronal and sagittal planes). Evaluation includes a quantitative assessment of spinal nerve root sizes (nerve root diameter immediately next to the ganglion, measured as coronal plane height with a cut-off value of >5 mm) or semi-quantitative scoring of abnormalities of the spinal nerve roots and trunks as normal, possibly abnormal or abnormal [20]. 

A prospective study of 32 CIDP patients and 22 non-CIDP patients evaluated the brachial and lumbosacral plexus, reporting a sensitivity of MRI in diagnosing CIDP of 81.25%, specificity of 68.18%, positive predictive value of 78.79%, and with negative predictive value of 71.43% [27]. 

Note: Before concluding that ultrasound or MRI abnormalities support CIDP, there should be no laboratory or clinical features suggesting other diseases, such as MMN, demyelinating CMT, IgM paraproteinemic neuropathy (especially with anti-MAG antibodies), POEMS syndrome, diabetic radiculoplexus neuropathy, amyloid neuropathy, neuralgic amyotrophy, leprosy, neurofibromatosis, or neurolymphomatosis [3].

CSF Analysis 

The 2021 EFNS/PNS guidelines suggest not performing CSF analysis if diagnostic criteria are already met. CSF analysis should be considered to exclude other diagnoses or to support the diagnosis of CIDP in the following circumstances: patients fulfilling diagnostic criteria for a possible CIDP but not CIDP; in cases of acute or subacute onset; and when an infectious or malignant etiology is suspected or possible.

High CSF protein levels are generally used as a positive indicator of CIDP. Levels ≥1 g/L strongly suggest CIDP, whereas levels below 1 g/L are more frequently associated with misdiagnosis [10]. Elevated protein levels in the CSF should be interpreted cautiously in patients with diabetes or those aged >50 years because these patients have higher than normal CSF protein levels per se [20,28]. 

New biomarkers, such as the dosage of sphingomyelin (SM) in CSF, have been studied, suggesting a promising application in evaluating response to therapy [29]. However, the 2021 EFNS/PNS guidelines are not included in the suggested workup.

*Nerve Biopsy* 

The 2021 EFNS/PNS guidelines suggest not performing nerve biopsy as a routine procedure to diagnose CIDP, but only in the following cases [3]: CIDP is suspected but cannot be confirmed with clinical, laboratory, imaging, and electrodiagnostic studies; and CIDP is suspected, but there is little or no response to treatment, such that an alternative diagnosis such as CMT, amyloidosis, sarcoidosis, or nerve sheath tumors/neurofibromatosis might be considered.

Nerve biopsies should be taken by skilled and specialized physicians and analyzed by expert pathologists. 

Immunological Testing

The 2021 EFNS/PNS guidelines strongly advise testing for monoclonal proteins in adult patients with a clinical suspicion of CIDP, with further evaluation recommended if a gammopathy is found. 

Notably, monoclonal gammopathy may be associated with neuropathies mimicking CIDP, such as anti-MAG IgM neuropathy, POEMS syndrome, multiple myeloma, or AL amyloidosis [3]. Anti-MAG antibody testing is advised in all patients with an IgM paraprotein fulfilling CIDP diagnostic criteria (predominantly distal CIDP) because a high titer of anti-MAG antibodies (>7000 Bühlmann Titre Units, BTU) would strongly imply a different diagnosis than CIDP [30].

Further, the 2021 EFNS/PNS guidelines suggest considering testing for nodal and paranodal antibodies in all the patients with clinical suspicion of CIDP when nodal and paranodal (anti-NF155, anti-CNTN1, anti-Caspr1) and possibly anti-NF140/186 antibody testing is available and meeting quality standards. It should also be considered to test in cases of resistance to standard therapy with IVIg and corticosteroids; in cases of acute or subacute aggressive onset and previous diagnosis of GBS or A-CIDP if a patient presents with low-frequency tremor, ataxia disproportionate to the sensory involvement, other cerebellar features, or predominantly distal weakness; in cases of respiratory failure and cranial nerve involvement; and in cases associated with nephrotic syndrome. It also occurs in patients with very high CSF protein levels.

**Figure 2:**
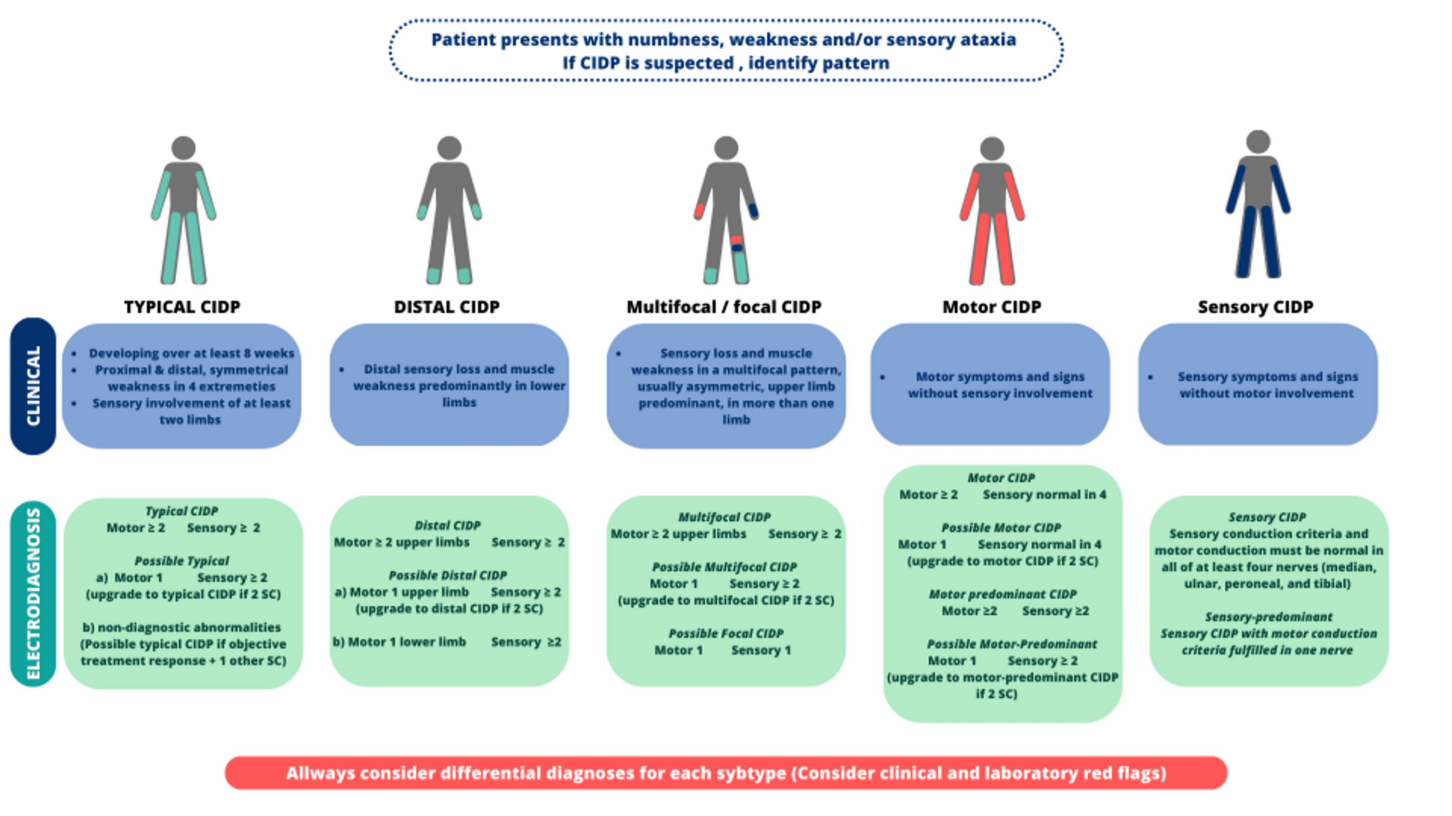
Overview of clinical and electrodiagnostic criteria for typical CIDP and atypical CIDP variants CIDP, Chronic inflammatory demyelination polyradiculoneuropathy; SC, Supportive criteria Figure credits: Rodolfo M. Roman-Guzman

Figure 2 provides an overview of each variant's clinical and electrodiagnostic criteria. 

Treatment 

CIDP treatment is initiated with one of the first-line therapies: immunoglobulins (including IV and subcutaneous routes of administration), corticosteroids, or plasma exchange [11].

Immunoglobulins 

Induction treatment: The total IVIg dose is 2 g/kg, divided over two to five days. However, not all patients respond to this first course, and two to five repeated doses of 1 g/kg IVIg every three weeks may be required before either the patient improves or it is decided that IVIg is ineffective.

Maintenance treatment: The most used regimen in clinical trials is 1 g/kg every three weeks, but in clinical practice, lower doses and more prolonged treatment intervals maintaining maximal sustained improvement should be considered (e.g., 0.4-1 g/kg every two to six weeks). If the patient is stable, it is recommended to evaluate periodically whether the IVIg dose can be reduced (e.g., by 25% per infusion) once every six to 12 months for the first two to three years of treatment, then less frequently (e.g., every 1-2 years). Subcutaneous human immunoglobulin (HIG) has been used successfully in treating CIDP with infusions at 35 ml/hr. In an extension of the Polyneuropathy and Treatment with Hizentra (PATH) study, doses of 0.2 g/kg or 0.4 g/kg weekly were used, and if clinically stable, the dose was switched to 0.2 g/kg weekly after 24 weeks [31].

Corticosteroids 

There are many regimens available, and there is no evidence to state that one is better than the other [3,4]. 

Starting doses [11]: Oral prednisone 1 g/kg/d to 1.5 g/kg/d or an alternate-day equivalent dose can also be used. Dexamethasone 40 mg/d can be taken four days every four weeks; another possibility is methylprednisolone 0.5 g one day each week or four consecutive days monthly. Reducing the corticosteroid dose should be attempted regularly to determine whether the current high dose is still required or whether the patient is in remission [3].

Plasma Exchange

Induction therapy: Within two to four weeks, 5-10 exchanges of 50 mL/kg plasma volume on alternate days are initiated [11]. 

Maintenance therapy: This includes one to three sessions every three to four weeks [11]. 

## Conclusions

This review comprehensively overviews the actualized diagnostic approach for CIDP diagnosis. Based on the evidence included, it is concluded that 2021 EFNS/PNS guidelines allow for a more accurate diagnosis and treatment of CIDP and its variants. New diagnostic tools and molecular approaches help in the diagnosis process but do not replace clinical and electrodiagnostic criteria. Knowledge of CIDP variants and specific differential diagnoses for each syndrome empowers clinicians for better practice.
